# Qualitative Study on Emotional Experiences and Coping Strategies in Patients With COVID-19 During the Early Stage of Wuhan Crisis

**DOI:** 10.1155/2024/6696049

**Published:** 2024-09-24

**Authors:** Junyao Li, Huirong Luo, Wenli Tang, Hong Qian, Huiping Yang, Qinghua Luo

**Affiliations:** ^1^Department of Psychiatry, The First Affiliated Hospital of Chongqing Medical University, Chongqing 400016, China; ^2^Hubei Dawu County Hospital of Traditional Chinese Medicine, Xiaogan, Hubei, China

**Keywords:** COVID-19, emotional experience, isolated inpatient, qualitative research, survivors

## Abstract

**Objective:** In the early stage of COVID-19 pandemic from December 2019 to March 2020, COVID-19 patients endured huge mental stress combined with constant physiological suffering. We aimed to summarize the emotional experiences of patients with COVID-19 during the early stages of the Wuhan crisis and present the coping strategies they used during the extreme time.

**Methods:** We did a qualitative study using an empirical phenomenology approach. COVID-19 patients with recovery and near discharge were recruited from the Dawu County Hospital of Traditional Chinese Medicine in Hubei province using purposive sampling. Semistructured face-to-face interviews were conducted by frontline medical staff and recorded by video and audio, then transcribed by two researchers separately. The Haase adapted version of the Colaizzi method was used to analyze the transcriptional data.

**Results:** We included 18 adult survivors of COVID-19 (33% female, 67% male) within the age range of 27–83 (mean age 48), and the average duration of isolation was 31.17 days. In conjunction with clinical data, we meticulously delved into the emotional trajectory of each survivor, spanning from the onset of illness through the phases of decline, improvement, and eventual recovery. Three theme categories were obtained from data analysis, including negative emotions and sources, coping strategies, and positive emotions and sources. COVID-19 patients adopted self-management strategies and received support from different roles when confronting high level of negative emotions.

**Conclusions:** Early survivors of COVID-19 experienced both negative and positive emotional experiences. Anxiety and other negative emotions were originated from both collective and individual concerns. The influence of the emotion sources differed at each stage of the disease. Patients coped with these stressors using external supports and self-adjustment. Still, comprehensive and targeted psychological services are needed.

**Summary**

• The study was carried out at the epicenter of COVID-19 during pandemic onset, thus bearing special and historical significance.
• It suggested necessity of comprehensive psychological evaluation and management when facing a new rising health crisis.
• The face-to-face interviews of early COVID-19 survivors guaranteed accurate, continuous, and detailed data about their psychological experiences.
• It identified various psychological stressors and supports behind emotional experiences, which may guide effective interventions in future crisis.

## 1. Background

**Figure 1:**
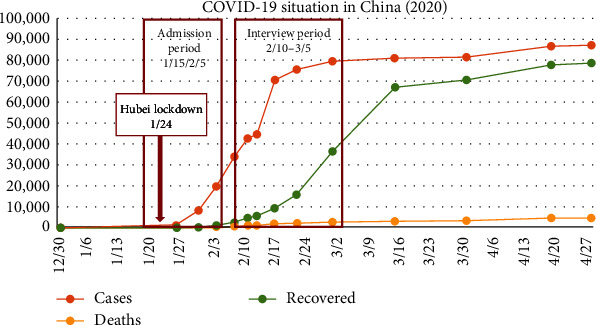
COVID-19 situation in China during the early stage (from Johns Hopkins University [6]).

Three years have passed since coronavirus disease 2019 (COVID-19) first emerged in Hubei, China [1]. Despite the reduced urgency due to effective treatments and widespread information, the darkest moments of the Wuhan outbreak, where the first case was identified in December 2019, remain unforgettable [2]. Severe acute respiratory syndrome coronavirus 2 (SARS-CoV-2), the virus causing COVID-19, spreads easily through direct contact, respiratory droplets, aerosols, and contaminated surfaces [3, 4]. With an incubation period of 3–14 days, confirming and managing infections are challenging [5]. On January 23, 2020, Wuhan implemented a lockdown due to the rapid spread of COVID-19 (refer to Figure 1) [7]. By March 11, 2020, the WHO declared this pandemic, with widespread cases both in China and internationally [8, 9, 10, and 11]. This pandemic has precipitated abrupt changes in individual, social, and economic facets of life [12, 13]. Owing to its elevated infectiousness, mortality, and persistent viral evolution, it has continually impacted daily life, instigating psychological stress, worry, and anxiety for all [14].

The primary manifestations of COVID-19 are focused on the respiratory system, with diagnosis confirmed through clinical symptoms like fever and fatigue, radiological evidence of pneumonia, and PCR testing [12, 15]. The disease exhibits distinct temporal patterns in symptom progression [16]. In Cluster 1, early symptoms peak and gradually subside, including runny nose, loss of appetite, and fever. Cluster 2 is characterized by stable symptoms like nausea, vomiting, chest tightness, and fatigue. Meanwhile, in Cluster 3, symptoms intensify sharply initially, then stabilize with slight variations over the next few months, encompassing palpitations, brain fog, and discomfort during movement. Beyond physical distress, patients often endure psychological suffering in this extreme circumstance. Respiratory viral illnesses have been linked to acute and long-term psychopathological outcomes in survivors, as evidenced by both the preceding severe acute respiratory syndrome (SARS) and middle east respiratory syndrome (MERS) outbreaks, both caused by coronaviruses [5, 17]. Survivors of SARS reported psychiatric symptoms, encompassing post-traumatic stress disorder (PTSD), depression, panic disorder, and obsessive–compulsive disorder (OCD), during follow-up periods spanning 1–50 months. Moreover, seropositivity for coronavirus has been linked to suicide and psychosis persisting for up to 1 year [18–20]. Survivors of COVID-19 have likewise reported elevated rates of PTSD, depression, anxiety, insomnia, and OCD, with incidence rates ranging from 10% to 35% [21]. Understanding the pathology associated with complex paroxysmal symptoms is critical for gaining deeper insights into their underlying mechanisms. Current neurobiological evidence has revealed psychiatric consequences of COVID-19 might arise from an immune response to the virus itself, inducing psychological stress and stress-related inflammation [22]. They may also potentially be caused by the infiltration of the neurotropic coronavirus into the brain, inducing neuronal injuries [23, 24]. Furthermore, various peripheral hormonal changes, including cortisol, can influence neurochemical alterations, thereby affecting mood-related activities [24].

Besides the evidence discussed earlier, various psychosocial stressors also contribute to psychopathology, leading to new symptoms or exacerbating existing mental or neurological issues [25]. Since the beginning of the pandemic, there has been rapid progress in establishing a connection between COVID-19 and mental health. Unique stressors related to COVID-19 encompass concerns about the illness, disruptions to daily life, fear of increased economic burdens, apprehension about social exclusion or quarantine, the loss of loved ones, feelings of powerlessness, boredom, and loneliness due to isolation [26, 27]. Stringent quarantine policies during the pandemic have led to insufficient or lacking psychological interventions for patients and medical staff, further escalating the prevalence of negative emotions [28]. A recent comprehensive meta-analysis on the prevalence of mental health problems discovered that patients with COVID-19 are prone to high levels of PTSD and sleep problems, followed by depression, anxiety, and stress symptoms [29]. Nevertheless, most individual studies are cross-sectional in design and lack longitudinal data from prepandemic to pandemic periods, making it challenging to establish causal relationships between mental impairments during the pandemic and long-term psychological health impacts in the postpandemic era [30]. Even when multiple assessments are conducted during the pandemic and prepandemic periods in surveys, the factors influencing mental health outcomes are frequently not based on structured clinical interviews. They are excessively confined to the impact factors within a limited scope and often lack assessments of methodological quality or bias risk in primary studies [31, 32]. Consequently, there is a notable high degree of heterogeneity in outcomes, leading to conflicting conclusions.

Given the limitations of quantitative research and the unique nature of the virus and its long-term effects, it is essential to involve stakeholders and incorporate the experiences of those affected. Accordingly, qualitative research designs have been employed to achieve a more comprehensive understanding. By placing the subject's perspective at the forefront, this approach explores their genuine inner feelings and opinions. Currently, the most recent qualitative investigations on COVID-19 involve retrospectively enlisting diverse groups (comprising the general public, healthcare workers, the elderly, parents, students, teachers, leaders, and more) and having them recall their experiences during the pandemic or postpandemic period, concentrating on aspects like mental health, education, public health infrastructure, and healthcare management [33–39]. In the postpandemic era, it is unquestionably more beneficial to shift the focus to the broader societal impact of the pandemic; however, this approach overlooks COVID-19 survivors who persist in experiencing long-term effects even after recovering. There have been fewer studies that have examined the psychological experiences of COVID-19 patients [40]. Limited data have permitted only a few qualitative descriptions during the early stages of COVID-19 and at the epicenter of this crisis. Historically, descriptive studies on patients' emotional experiences have yielded positive outcomes. However, relying on online or diary-based methods has distanced researchers from face-to-face investigations and clinical environments [40–42]. In this way, many researchers have a limited understanding of the clinical conditions on the frontline of the pandemic, potentially introducing biases in research design and conclusions. Specifically, these studies overlook the comprehensive consideration of clinical data, hindering a thorough exploration of emotional occurrences, and experiences aligning with the clinical course. There still lacks the detail that provides thorough longitudinal insight from patients.

In conclusion, numerous studies indicate that the long-term mental health of COVID-19 survivors is adversely affected by issues such as depression and high levels of post-traumatic stress. The significance of mental health during the COVID-19 pandemic extends beyond impacting illness progression and recovery; it also shapes outcomes in social and personal functioning. Undoubtedly, it can amplify the efficacy of both individuals and the social milieu, playing an indispensable role in ensuring the vitality and efficiency of any society [43]. Identifying determinants of compromised mental health, alongside those fostering positive emotions, becomes imperative for enhancing our understanding of the pathology, and formulating early preventive strategies aimed at mitigating psychological harm or trauma, while concurrently nurturing a supportive and cohesive social milieu. A nuanced comprehension of these factors holds the potential to significantly guide us through the challenges presented by the pandemic and advance the collective well-being.

Psychosocial data relevant to individuals living with COVID-19 is worth further study to enhance interventions and address the psychological impact of the COVID-19 pandemic [44]. To address the gap in scientific reports regarding the emotional impacts of the COVID-19 pandemic during its early stages and to gain insight from the subjective perspective of recovered hospitalized COVID-19 patients, this study employed an empirical phenomenology method of qualitative analysis. Eighteen survivors were interviewed prior to their discharge in the second month of the COVID-19 outbreak. Our objectives were as follows: (1) to summarize the emotional experiences and sources of patients with COVID-19 during the early stages of the Wuhan crisis; (2) to present the coping strategies they used during the extreme time.

## 2. Methods

### 2.1. Study Design and Participants

We conducted a qualitative study that included individual face-to-face interviews, employing an empirical phenomenological research approach, which is a qualitative research method [45]. Empirical phenomenology aims to describe the daily life experiences of individuals and the meanings these experiences hold for those who live through them [46]. Essentially, it focuses on describing commonalities in the experiences of a group. We selected this approach for our research to unveil the emotional experiences of COVID-19 survivors during their hospitalization and as they approached discharge amid the COVID-19 outbreak in Hubei, China. We analyzed both video and audio recordings of survivors' interviews and observed their body language to ensure consistency with their statements. Additionally, we used repetition, detailed inquiry, and rhetorical questioning to validate interview information when necessary.

We employed the purposive sampling method to select 18 respondents from COVID-19 patients hospitalized at the Dawu County Hospital of Traditional Chinese Medicine in Xiaogan, Hubei, which is situated ~60 km away from Wuhan. We included COVID-19 survivors who had recently recovered and were about to be discharged as inpatients, willing to participate in this study with informed consent, and with no impairment of speech, consciousness, or cognitive function. Participants who spoke only in a regional dialect that we could not understand were excluded. To ensure that emotional experiences were caused by COVID-19, we also excluded individuals with previously diagnosed mental illness or underlying conditions. The inclusion and exclusion criteria are presented in Table 1. The research adheres to the principles outlined in the Helsinki Declaration. Ethics approval for this retrospective research was subsequently obtained from the institutional review board at the First Affiliated Hospital of Chongqing Medical University (2023-K023).

### 2.2. Procedures

This qualitative study employed data collected between February 10 and March 5, 2020. During this period, we conducted interviews with 18 COVID-19 survivors who had been transferred to the Dawu County Hospital of Traditional Chinese Medicine for isolation and treatment at different times, spanning from mid-January to early February. Video and audio data were acquired through semistructured in-depth interviews conducted for clinical purposes. The interviews, each lasting 10–20 min, included clear and comprehensible questions. The interview outline was collaboratively developed by frontline psychiatry professionals and nursing personnel, taking into account the clinical course, psychological health status, and patient feedback. Questions explored: (1) emotional experiences at different stages of the disease course; (2) sources of the patient's emotions; and (3) methods employed by the patient to regulate their emotions. It included the following key questions: (1) “When did you first start to feel unwell? What were your symptoms?” (2) “How did you feel at the worst point of your illness?” (3) “What was your lowest emotional state during that time? What did you do to relieve it?” and (4) “Is there anything you would like to convey to our doctors, nurses, or fellow patients?” To obtain detailed descriptions, open-ended follow-up questions were used. These included inquiries such as: “When was the most challenging moment for you?”, “What was your greatest source of concern during that period?”, “Is there a message you wish to convey to others?”, “Whom else would you like to express gratitude to?”, and “Is there anything else you would like to share?”. These follow-up questions aimed to enhance the depth of the interviews.

To prevent the spread of the virus, comprehensive epidemic prevention measures were implemented both before and after the interview. Respondents were required to wear an N95 face mask during the interviews. The medical staff wore comprehensive epidemic prevention suits, comprising disposable medical gowns, N95 face masks, face shields, disposable medical gloves, disposable medical headsets, and disposable medical shoe covers. Both parties were positioned at a 120° angle from each other and maintained a linear distance of 0.5 m. The camera was positioned ~2 m in front of the respondents, and they were instructed to face it while speaking. After each interview, the equipment, tables, chairs, medical staff, and interviewees' clothing, as well as the surrounding area, were disinfected with a 75% alcohol concentration.

To supplement the information regarding age, sex, family situations, admission and discharge dates, and length of hospitalization, medical charts were reviewed for all respondents. Our team consisted of nurses and psychologists who were directly involved in the patients' care throughout their stay (the qualifications of the members are detailed in Table 2). All interviews and analyses were diligently carried out by the corresponding author (QHL), and scheduled during break times with the assistance of clinical medical staff (WLT, QH, or HPY) to guarantee privacy and a quiet environment, free from external interference. The respondents were informed of the interview's purpose, and a positive relationship was cultivated between the interviewer and the respondents. Primarily through one-to-one face-to-face conversations, respondents were initially invited to introduce themselves using the guiding words, “Please share your emotional experiences from the onset of your illness to recovery.” The interviews were concluded upon confirming that all relevant content had been gathered, and data saturation was achieved. Specifically, when the data collection process revealed no new information and redundancy began to emerge, the researchers (JYL, HRL, and QHL) collectively reached a consensus to cease further data collection. Eventually, data saturation was achieved with 18 patients (*N* = 18). Following the interviews, two researchers independently transcribed the video and audio recordings into text. The two transcripts were reviewed, and any disputed sections were relistened to verify their accuracy.

### 2.3. Data Analysis

The Haase adapted version of the Colaizzi method was used to analyze the transcriptional data [48, 49]. The analysis entailed reading the interview records multiple times to grasp the conveyed meaning, identifying key phrases, reformulating them in a general manner, and developing and validating the meaning through discussions within the research team to achieve a consensus [50]. To minimize the impact of personal values in data analysis, two authors (JYL and HRL) independently summarized noteworthy statements and coded recurring meaningful perspectives. They then determined common themes through cross-checking and discussion to mitigate bias and sought third-party opinions in the event of disputes. We identified and organized topics into themes and clusters and provided a comprehensive description of the topics.

## 3. Results

Our sample consisted of 18 COVID-19 survivors aged 27–83 (mean age 48). Among them, 12 were females, and six were males. General information about all respondents is provided in Table 3. P3 and P4 were a couple, while P6, P7, and P8 were from the same family: a younger brother, an older brother, and their father. The duration of isolation they spent in the ward before the interviews ranged from 27 to 38 days, with an average length of 31.17 days. The interviews ranged from 10 to 20 min, with an average duration of 13.4 min.

We categorized the emotional experiences of survivors into three themes: “negative emotions and sources,” “coping strategies,” and “positive emotions and sources” (see Table 4 for the framework of themes and clusters).

### 3.1. Theme 1: Negative Emotions and Sources—Inevitable Bad Feelings and Stress When Facing the Unknown Infectious Disease

#### 3.1.1. Negative Emotions

Many survivors reported heightened negative emotions following infection, particularly during the early phases. These emotions encompassed worry, fear, stress, tension, irritability, depression, despair, and among others. The most frequently cited negative emotion was worry, concerning both their own well-being and that of their acquaintances (*n* = 10). For example, one participant expressed, “I harbored concerns that there was no chance for survival” (P4), while another mentioned, “I just worried about my family and if they were infected” (P11). The second most frequently mentioned emotion was fear (*n* = 6). Patients harbored fears regarding the worsening of the disease and the prospect of death. A participant conveyed, “This being an unknown disease made me very afraid” (P3), and another said, “I had a strong fear of death when I experienced great pain during the illness” (P17). The third most frequently cited emotion was stress (*n* = 4). One participant shared, “My wardmates were dying, making me very stressed” (P3), and another mentioned, “My psychological pressure was extremely high after I was transferred to the critical unit” (P2).

#### 3.1.2. Sources of Negative Emotions

Survivors provided detailed insights into the origins of negative emotions, categorizable into collective and individual sources. Collective sources included external information related to the illness, daily routines during isolation, and the witnessing of death and suffering.

External information about the disease and the ongoing pandemic had a significant impact on patients' emotions. During that time, there was limited knowledge about the COVID-19 virus and its potential clinical and pandemic implications. One patient expressed, “Being a new disease without any medication made me very afraid, and I had concerned that survival was not possible. I felt desperate” (P4). Another patient mentioned, “Many people were reported to be dying in the daily news, and the lack of a vaccine disturbed me” (P16).

The daily routines during hospitalization could act as a stressor, especially when patients observed severe symptoms in others and faced an unfamiliar environment. A patient stated, “Upon learning about the tremendous efforts of the doctors and nurses and the severity of symptoms in other patients, I experienced significant discomfort and sadness” (P18). Additionally, all patients were isolated in an unfamiliar hospital environment, lacking sufficient living space, and were unable to receive visits or care from family members. This situation led to feelings of boredom and discomfort in most patients. For example, one patient expressed, “During the second week of hospitalization, I felt significant boredom and a desire to leave” (P6).

Moreover, witnessing the suffering and mortality of fellow patients increased the psychological burden on survivors. A patient who spent 8 days in the intensive care unit noted, “There were constant deaths in this ward. It caused significant stress for me” (P3).

Individual sources of negative emotions primarily revolved around physiological pain, disrupted life, and family difficulties, which significantly impacted the mental well-being of patients. Physiological pain was defined as any subjective discomfort experienced by patients after contracting COVID-19, as indicated by the interview content. This discomfort spanned various sensations, such as abdominal discomfort, headaches, sore throat, lung pain, body aches, temperature fluctuations, and muscle soreness. The intensity of physiological pain could serve as an indicator of the severity of the illness. As patients' symptoms improved, their discomfort and negative emotions also diminished. For instance, one patient recounted, “The worst time was when I had a high fever that lasted for several days, making me think that my life was in danger. I was on the brink of complete despair” (P5). Another patient described, “I suddenly felt cold, so I took my body temperature and discovered I had a high fever. I became nervous all of a sudden” (P13). A different patient shared, “I was completely unable to take care of myself due to constant nausea. At that time, I wanted to give my last wishes to my son” (P14). Advanced age, a history of previous surgery, and pre-existing health conditions appeared to weigh heavily on patients' minds. One patient expressed, “I'm 83 years old, and my biggest fear was that I was too old to make it through this time” (P12), while another said, “I had heart surgery before, so I was afraid I couldn't make it” (P3).

Patients also expressed concerns about their families. The COVID-19 outbreak coincided with the Chinese Spring Festival, a time when people traditionally travel and gather, increasing the risk of clustered infections. Numerous patients were concerned about the potential transmission of the virus to their family members and felt a sense of responsibility for their well-being. Some patients also fretted about unfinished personal and family matters. “Having recently visited my relatives during the Spring Festival, I never anticipated that my entire family would contract the virus from me. Nevertheless, circumstances unfolded in this manner. I consistently held myself accountable and experienced a deep sense of guilt” (P7). “The worst period for me was when I didn't know if my family had been infected by me because, at that time, there were eight people in my family, and my sister-in-law's child was only 2 years old” (P11). “I worried about the elderly and children in my family. Additionally, there was a lot of household work that had not been completed” (P16).

### 3.2. Theme 2: Coping Strategies

Each participant shared their coping mechanisms for addressing negative emotions, deriving inspiration from their determination to survive and the perception of being indispensable to others. The coping mechanisms could be categorized into two clusters: “self-comfort” and “diverting attention.”

#### 3.2.1. Self-Comfort

Survivors utilized self-comfort techniques to soothe themselves and cultivate confidence in overcoming the illness (*n* = 16). They endeavored to persuade themselves that COVID-19 was a controllable illness. While some downplayed the gravity of their infection, others acknowledged it and refrained from overthinking, and a few even established precise time constraints for worrying. “I consistently remind myself that it's merely a minor ailment, and as a result, my mood significantly relaxes… Don't overly stress about it. It's akin to a common cold” (P1). “Numerous individuals are undergoing the same suffering as me. I'm neither unique nor distinct” (P4). “Embracing the reality, excessive contemplation is futile. My sole recourse is to earnestly collaborate with the prescribed treatment” (P7). “I instituted a 5-day observation period for myself. Following this duration, I perceived no deterioration. Subsequently, I experienced a substantial relaxation” (P13).

#### 3.2.2. Divert Attention

Some patients redirected their attention by engaging in relaxing activities such as chatting, watching, listening, exercising, and reading (*n* = 9). They did these activities either alone or together with their wardmates. “I would chat with my wardmates, which relieved my nerves” (P1). “When my breathing wasn't too bad, I listened to songs, watched TikTok, and danced in the ward, which greatly improved my state of mind” (P2). “I watched TikTok and movies, listened to music, and chatted with my family. These activities gradually improved my mood” (P15).

### 3.3. Theme 3: Positive Emotions and Sources—Patients Derived Encouragement and Support From Various Roles in Their Lives

#### 3.3.1. Positive Emotions

Individuals with COVID-19 experienced a sense of upliftment and received encouragement both during the treatment process and in the recovery phase. They frequently expressed feelings of confidence, belief, trust, encouragement, and relaxation, which increased as the treatment progressed. “As symptoms gradually alleviated each day, my emotional state improved accordingly” (P1). “I maintain confidence and composure throughout my hospitalization” (P10). “I encourage myself every day. My child is still young, and I must live well and be happy every day” (P2). “I am aware that the virus itself is not inherently lethal, and I hold the belief that I will recover” (P7). “The healthcare professionals in this facility make utmost efforts to provide encouragement and attend to my needs, leading to a gradual calming of my emotional state” (P4).

#### 3.3.2. Sources of Positive Emotions

Families played a pivotal and supportive role throughout the hospitalization period. On one hand, patients received encouragement from their family members. For instance, one patient shared, “When I talked to my family on the phone, they encouraged me and told me to treat it as an ordinary illness, and everything will be fine” (P1), and another mentioned, “They told me not to be afraid on video calls” (P5). Additionally, some patients who were hospitalized alongside their family members found mutual support in each other. One patient described, “My wife and I were infected at the same time, and we were isolated in the same hospital. We met each other when we fetched water. Then we talked about the situation and encouraged each other” (P13). On the other hand, some patients felt a sense of responsibility to support their families economically or to take care of the elderly and young members, which made them feel constantly needed. One patient expressed, “For my wife and two children, I am the breadwinner of my family… My health is crucial. I won't give up” (P5).

Patients also found encouragement from the medical staff through supportive words and behaviors, contributing to emotional stability and positive feelings. For example: “In the second re-test when I received a positive PCR result, I felt heavy with suspicion of disease recurrence. Later, the doctor told me it was only because of the residual virus in my body. Then I gradually calmed down” (P1). “I believe the doctors' encouragement and the little white lies they told me are very important, as they gave me great confidence” (P2). Medical staff not only provided psychological support but also exhibited a strong sense of responsibility in their daily practices, serving as significant motivation for patients: “I was touched when the nurses used a basin to catch stool and urine for severe cases, just like they were helping their own family. I trust them very much” (P14). “Doctors and nurses made daily rounds in the ward, asking if we felt uncomfortable or if we needed anything, which made me feel very reassured” (P17). “I wasn't scared. I thought, with so many good doctors in the hospital, nothing bad will happen to me” (P18).

Interactions with wardmates also generated positive emotions. Shared experiences were common conversation topics, providing mutual encouragement. For example, one patient expressed, “We happily talked about everything with each other in the ward that we forgot the pain and became filled with positive energy” (P5). Another patient mentioned, “Seeing other patients in the same ward, I gradually accepted the fact that I was infected, and I adjusted my mindset gradually” (P6).

Media and beneficial policies played essential roles in spreading positive information among patients. One patient stated, “It's very helpful to learn through mobile phones to improve my understanding of the disease and enhance my confidence” (P13). Another mentioned, “I watch the news on my phone every day and have great confidence in my country. After all, we have successfully overcome big problems like SARS” (P16). “Don't worry, we are under state-controlled treatment” (P10). “I am confident that we will not be defeated by any difficulties with a good national policy and the government's correct guidance” (P16).

Finally, as patients' symptoms gradually improved and they received clinical evidence of recovery, such as negative PCR results, decreased CT problems, and information about impending discharge, their emotions began to ease. Patients often experienced a sudden shift in mood. One patient shared, “This morning, I found out that I am going to be discharged, and suddenly I felt relaxed without any pressure” (P9). Another mentioned, “After three CT scans, my worries disappeared when the doctor said my symptoms hadn't worsened” (P12). Less severe symptoms also reduced the psychological burden. One patient stated, ”There was nothing to worry about because my symptoms weren't that bad” (P15).

## 4. Discussion

With the gradual normalization of pandemic prevention and control measures, it becomes historically significant to reflect on the emergence of COVID-19. During that time, our country implemented strict isolation policies. Infected patients were hospitalized without the company of their family members and were unable to step out of their wards. Their daily lives were completely disrupted due to the city-wide lockdown. Even with free treatment policies and nationwide support, the overwhelming stress and adaptation difficulties were clearly evident among patients dealing with both the microvirus and macrolife uncertainties [51]. A survey of 1210 people in China found that 53.8% of them had moderate to severe psychological damage in 2020 [51]. COVID-19 patients were the first group to experience physical pain and psychological problems, with residual psychological issues persisting even after recovery [52]. Mental health issues in the postillness period have emerged as essential health concerns. Undoubtedly, there is a pressing need to enhance our understanding of the pathology to facilitate the design and development of safe and tailored support services and approaches for long-term COVID-19 patients. However, due to limited access to inpatients at that time, the detailed emotional experiences of the first batch of infected/recovered patients are yet not sufficiently described. Our study employed a qualitative method and integrated relevant clinical data to provide a comprehensive description of the psychological experiences and coping strategies of patients from the onset of the disease to recovery during the initial phases of the Wuhan crisis. On the one hand, a comprehensive summary and understanding of the emotional processes of survivors during the pandemic are crucial for early identification of signs and symptoms of psychological distress. On the other hand, exploring emotional sources and coping strategies throughout the disease course guides clinical decisions, developing effective long-term support. This insight also helps explore interventions for evolving mental health challenges in future pandemics, improving mental health services. Additionally, it sheds light on stressors contributing to mental symptom during pandemic and residual psychological issues in postpandemic period, aiding in the understanding of the psychopathology of psychiatric consequences.

The psychological state of COVID-19 patients is not optimistic. This study integrates the clinical experiences of frontline personnel and medical records to provide a comprehensive depiction of the emotional trajectory of COVID-19 survivors at the epicenter of the pandemic's emergence and during its initial stages. Three overarching themes surfaced from the data: “negative emotions and sources,” “coping strategies,” and “positive emotions and sources.” It was observed that prevalent negative emotions, such as anxiety, worry, and fear, were particularly prominent in the early and middle phases of the illness. These emotions emanated from collective sources, including external information about the illness, disrupted routines, and witnessing death and suffering. Individual sources of negative emotions encompassed physiological pain, disruptions in daily life, and family challenges. The pandemic not only imposes physical harm on patients but also induces sustained mental health repercussions due to various stressors, a fact that should not be underestimated. The study underscores the profound impact of negative external information on patients, emphasizing the imperative need to institute national policies in support of patients and to monitor information from external media in real time to ensure that patients receive accurate and favorable information at an early stage. Additionally, the study advocates for the early implementation of psychological interventions, such as mindfulness and compassion-based psychotherapy, which have demonstrated effectiveness in significantly mitigating anxiety and stress levels in patients' mental health during COVID-19 lockdowns [53]. Furthermore, peer education and psychological support are identified as effective strategies for alleviating anxiety, depression, disease-related pain, and sleep issues among patients. These approaches furnish a valuable support system and instill a sense of understanding and empathy, significantly enhancing patients' mental well-being in challenging times like the COVID-19 pandemic [54].

Although daily life has now returned to normal, individuals may experience unsettling overwhelming sensations and thoughts associated with the event, leading to flashbacks or nightmares. Furthermore, various psychological distresses unrelated to the traumatic event have also emerged. Numerous studies emphasize the lasting detrimental impact of mental health problems and have documented that COVID-19 survivors face an increased risk of experiencing mental health issues, both in the aftermath of the illness and over the long term. In psychiatric outpatient settings following the lifting of lockdowns, individuals who have recovered from COVID-19 often report new-onset psychological problems, including depression, anxiety, intrusive thoughts, insomnia, and, in some cases, delirium, psychotic symptoms, and even thoughts of suicide [17]. A recent meta-analysis involving over 1.28 million COVID-19 patients across 32 countries revealed that within 12 months postinfection, 50.1% of recovered individuals experienced at least one sequela, and 19.7% exhibited at least one mental health symptom, including depression, PTSD, anxiety, and insomnia, with occurrence rates ranging between 10% and 20% [52]. The PHOSP-COVID-19 project at the University of Leicester in the UK, which tracks survivors' health for 6 months postrecovery, found that over 25% experienced symptoms of depression or anxiety, and 12.2% of the recovered individuals had PTSD [55]. Similarly, a retrospective study found that 2 years after recovering from COVID-19 infection, survivors had a higher risk of neurological and psychiatric disorders, such as dementia, psychotic symptoms, and seizures, compared to other respiratory disease survivors [56]. Survivors of COVID-19 have undergone various severe psychological issues and experienced significant traumatic stress reactions. Even after recovery, all patients infected with COVID-19 are considered to be in a catastrophic situation [25]. A variety of factors contribute to this situation, but current research is primarily concentrated on investigating neurobiological evidence, suggesting that coronaviruses can lead to psychopathological sequelae through direct infection of the central nervous system or indirectly via immune responses [18, 28]. It is important to note that psychological factors during the COVID-19 pandemic also play a significant role in psychiatric consequences [57, 58]. The psychological stressors we investigated, including collective (external information about the illness, daily routines during isolation, and witnessing death and distress) and individual (physiological pain conditions, disrupted daily life, and family difficulties) factors, may interact to define psychopathological outcomes. In conclusion, these stressors not only impact mental health during COVID-19 but also contribute to residual psychological problems or trauma after recovery.

To cope with their negative emotions, patients typically employed strategies centered around self-comfort and attention diversion, while receiving social support mainly from medical staff and family members. However, the study showed less evidence of self-coping strategies and more evidence of the indirect influence of individuals' personal resilience to the disease through external positive sources. The interviews revealed that patients primarily derived positive emotions from interactions with various individuals and entities. These positive emotions included relaxation, trust, confidence, encouragement, and gratitude, especially after recovery. These positive sources of emotion came from family members, medical care providers, wardmates, media, national policies, and a decrease in symptom severity.

The study underscores the significance of fostering communication and trust among patients, medical staff, and family members during hospitalization. Moreover, ensuring symptom relief, access to positive external information, and the implementation of supportive national policies play pivotal roles in providing essential support for patients on their path to recovery and emotional well-being. In both pandemic and postpandemic times, it is essential to uphold a positive social media environment, utilize online platforms for mental health education and consultation, increase government support for mental health system development, and strengthen the workforce for mental health services, aiming to enhance the overall level of emergency psychological intervention during public health events.

**Figure 2:**
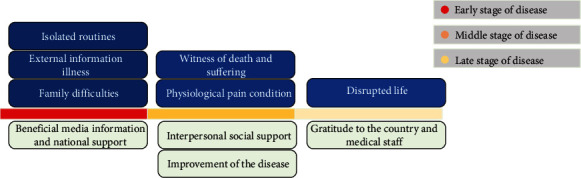
Major influence sources in different disease course.

The influence of the sources mentioned above varies at different stages of the disease course, as illustrated in Figure 2. In the early stage, patients' positive and negative emotions are strongly influenced by national policies and media. Patients experience more negative emotions due to isolated routines, external information about the disease, and family difficulties during this period. In the medium term, patients receive more positive feedback from interpersonal support, including family, medical staff, and fellow patients, as well as improvements in their disease. However, patients may still feel anxious and fearful when their symptoms have not been relieved or when they witness a fellow patient's serious illness or death. In the later stage, patients primarily express gratitude toward the country and the medical staff. Nevertheless, they may also worry about the long-term impact of COVID-19 on their daily lives. These findings emphasize the importance of implementing comprehensive intervention and positive mood promotion programs throughout the entire course of the disease. It is need to note that the influence of a particular source may vary in intensity but is not absent at other times during the disease course.

The results of our study carry significant implications for the psychological assessment and treatment of individuals affected by the infection. Confronting novel health crises necessitates healthcare systems to attend to not just the physical but also the psychological welfare of individuals, ensuring holistic care. This approach has been underscored in prior health crises, including the Ebola epidemic and the SARS outbreak [59–61]. Throughout the research process, frontline psychological service providers suggested various strategies to aid patients. These strategies encompassed encouraging patient communication, fostering emotional interaction between patients and clinical staff, broadcasting messages from recovered patients, and organizing uplifting moderate exercise activities within the wards. It appeared to be beneficial in increasing social support and reducing feelings of isolation among patients [62]. Nevertheless, there might be additional measures that can be implemented to support patients during health crises like this. This might involve extensive communication and education about the disease, implementing systematic mental health evaluations, providing accessible individual psychological consultations, offering provisional medical interventions for conditions like insomnia, and creating a healthcare environment with more extensive activity space and improved privacy to assist patients in restoring their customary living conditions [40]. Apart from psychological interventions, it is crucial to acknowledge and tackle the sources of negative emotions, such as physical discomfort, incorrect or insufficient information, reduced communication, and living issues. Dealing with these underlying issues can likewise exert a significant influence on the well-being of individuals during health crises.

Our study represents the inaugural qualitative exploration of the emotional experiences of COVID-19 survivors during the early stages of the pandemic, situated at its epicenter. By drawing on the clinical experience of medical frontlines, we have developed a comprehensive interview protocol specifically tailored to address the challenges posed by the pandemic crisis. Prior to the interviews, these patients had recently confronted the difficulties of hospitalization due to COVID-19, navigating significant psychological burdens arising from extreme physical, individual, and social conditions. The emotional experiences, as articulated during the interviews, exhibit a remarkable consistency from the time of infection to recovery, providing a more systematic and objective insight into their emotional journey. The value of these data is heightened by our use of face-to-face interviews on the frontline, ensuring accuracy and minimizing memory bias or incomplete information that may occur in remote online or telephone interviews. While the unique circumstances of our study may pose challenges for replication, our findings align with previously published studies in Germany, the United States, Ghana, and Iran, offering additional support for the consistency of our results [40, 59, 63, 64]. Compared to the existing literature, our study emphasizes the significant impact of external sources on positive emotions, with less evidence of self-coping strategies. This contrasts with previous studies that highlight personal resilience in coping. We found that national policies and media communication strongly influenced both positive and negative emotions, especially in the early stages of the disease. Additionally, survivors in our research mentioned loneliness less often, likely due to support from external factors like the media, fellow patients, and healthcare professionals. This underscores the crucial role of social support and information exchange in reducing psychological distress. Moreover, our study introduces a chronology of the extent of influence from these sources, a topic that has not been extensively explored in prior research, contributing to a comprehensive understanding of the emotional experience throughout the course of the illness. Simultaneously, existing studies have primarily focused on the mental health of patients during the disease course, neglecting to delve into the repercussions on mental health postrecovery. Indeed, it is crucial to contemplate the possibility that psychiatric symptoms encountered by COVID-19 survivors might stem from traumatic stress responses or residual effects of the illness itself [65, 66]. This recognition can contribute to providing appropriate interventions and support to address the mental health needs of these individuals, both during and after their recovery.

Nevertheless, there are also some limitations. Sample inclusion cannot be fully generalized to all patients and regions due to containment policies, the long duration of the disease, and limited assistance. We only focused on a specific population in a specific geographical location, which limits the transferability of the findings to other contexts. As respondents were closely at discharge with favorable treatment efficacy, answers about medical services tend to be positive when interviewed face-to-face by researchers, who also belonged to frontline clinical staff. Furthermore, our respondents were treated at the same hospital with similar treatment schedules, which might have made their treatment experiences less diverse. Finally, our study lacks follow-up surveys in postpandemic. The later emotional experiences of survivors after discharge could not be illustrated, which is significant given that many patients today continue to experience psychological problems after recovery.

## 5. Conclusions

Patients with COVID-19 experienced increased anxiety and other negative emotions, stemming from both collective and individual concerns. To cope with these emotions, patients employ strategies of self-comfort and attention diversion, while seeking social support from various sources, including family, wardmates, medical staff, government policies, and the reduction of symptom severity. It is important to note that the influence of these emotional sources varies at different stages of the disease. This study provides a comprehensive description of COVID-19 patients' emotional experiences from the onset of infection to their eventual recovery. However, it is evident that there is still a significant demand for psychological services to address the emotional suffering of these patients. This highlights the need for enhanced psychological observation and intervention, as well as increased social support during the early stages of a health crisis.

## Figures and Tables

**Table 1:** Inclusion and exclusion criteria.

Inclusion criteria	Exclusion criteria
• COVID-19 survivors near discharge	• Survivors speak only regional dialects that researchers could not understand.
Diagnosis and discharge criteria based on *The third trial version of China's novel coronavirus pneumonia diagnosis and treatment protocol* [47].	• Previously diagnosed with mental illness.
-Diagnosis criteria: (1) sputum, throat swabs, lower respiratory tract secretions, and other specimens were tested for the presence of novel coronavirus nucleic acid using real-time fluorescent RT-PCR. (2) Viral genes that are highly homologous to known novel coronaviruses are present.	-Including mood disorders, schizophrenia, eating disorders, and substance use disorders.
-Discharge criteria: If body temperature returns to normal for more than 3 days, respiratory symptoms improve significantly, lung imaging shows clear resolution of inflammation, and two consecutive negative results from respiratory pathogenic nucleic acid tests (with an interval of at least 1 day), the patient can be discharged.	• Previous underlying condition.
• Voluntary participation.	-Chronic diseases: obesity, heart disease, cancer, hypertension, lung disease, and diabetes.
• No impairment of speech, consciousness, or cognitive function.	

**Table 2:** Qualifications of research members.

Name	Qualification
Qinghua Luo	Male. Chief physician of the First Affiliated Hospital of Chongqing Medical University. A psychiatrist with over 20 years of experience in psychiatry. Made major contributions to the fight against COVID-19, awarded “Psychiatrist with Special Anti-COVID-19 Contribution” by the Chinese Psychiatrist Association.
Huiping Yang	Female. Supervisor nurse of the Dawu County Hospital of Traditional Chinese Medicine with 20 years of nursing experience. A caregiver on the frontline in the early stages of COVID-19.
Junyao Li	Male. Postgraduate student of psychology at Chongqing Medical University, guided by QHL.
Huirong Luo	Female. Postgraduate student of psychiatry at Chongqing Medical University, guided by QHL.
Wenli Tang	Female. Supervisor nurse of the Dawu County Hospital of Traditional Chinese Medicine with 10 years of nursing experience. A caregiver on the frontline in the early stages of COVID-19.
Qian Hong	Female. Supervisor nurse of the Dawu County Hospital of Traditional Chinese Medicine with 9 years of nursing experience. A caregiver on the frontline in the early stages of COVID-19.

**Table 3:** Basic information of participants.

Number	Gender	Age (years old)	Length of stay (day)
P1	Male	27	22
P2	Female	34	34
P3	Male	76	34
P4	Female	70	35
P5	Male	29	25
P6	Male	41	33
P7	Male	41	33
P8	Male	69	34
P9	Male	32	25
P10	Female	55	27
P11	Female	34	31
P12	Female	83	38
P13	Male	38	29
P14	Male	58	30
P15	Male	32	26
P16	Male	55	37
P17	Male	33	32
P18	Female	67	36

**Table 4:** Themes and clusters.

Panel: themes and clusters
1. Negative emotions and sources—inevitable bad feelings and stress when facing the unknown infectious disease:
A. Negative emotions.
B. Sources of negative emotions—collective and individual.
2. Coping strategies:
A. Self-comfort.
B. Divert attention.
3. Positive emotions and sources—patients obtain encouragement and support from different roles:
A. Positive emotions.
B. Sources of positive emotions—family members, medical personnel, wardmate, media, national policy, and symptom severity.

## Data Availability

The data generated and analyzed during the current study are available from the corresponding author upon reasonable request.
